# 2-Methoxy­naphthalene-1-carbaldehyde

**DOI:** 10.1107/S1600536809014287

**Published:** 2009-04-22

**Authors:** Chunbao Tang

**Affiliations:** a Department of Chemistry, Jiaying University, Meizhou 514015, People’s Republic of China

## Abstract

In the title compound, C_12_H_10_O_2_, the aldehyde and meth­oxy groups are slightly twisted around the single bonds that join them to the naphthalene ring system. In the crystal structure, mol­ecules are linked through inter­molecular C—H⋯O hydrogen bonds, forming chains running along the *c* axis.

## Related literature

For crystal structures of Schiff bases, see: Yehye *et al.* (2008 ▶); Tabatabaee *et al.* (2007 ▶); Zhang & Li (2007 ▶). For bond-length data, see: Allen *et al.* (1987 ▶).
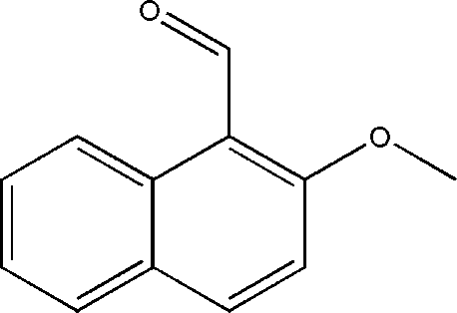


## Experimental

### 

#### Crystal data

C_12_H_10_O_2_
*M* *_r_* = 186.20
Monoclinic, 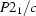

*a* = 8.689 (3) Å
*b* = 14.155 (4) Å
*c* = 7.667 (2) Å
β = 94.805 (4)°
*V* = 939.7 (5) Å^3^
*Z* = 4
Mo *K*α radiation
μ = 0.09 mm^−1^
*T* = 298 K
0.20 × 0.20 × 0.18 mm

#### Data collection

Bruker SMART CCD area-detector diffractometer
Absorption correction: multi-scan (*SADABS*; Sheldrick, 1996 ▶) *T*_min_ = 0.982, *T*_max_ = 0.984
5187 measured reflections
2046 independent reflections
1477 reflections with *I* > 2σ(*I*)
*R*_int_ = 0.018

#### Refinement

*R*[*F* ^2^ > 2σ(*F* ^2^)] = 0.043
*wR*(*F* ^2^) = 0.124
*S* = 1.03
2046 reflections
128 parameters
H-atom parameters constrained
Δρ_max_ = 0.12 e Å^−3^
Δρ_min_ = −0.17 e Å^−3^

### 

Data collection: *SMART* (Bruker, 2002 ▶); cell refinement: *SAINT* (Bruker, 2002 ▶); data reduction: *SAINT*; program(s) used to solve structure: *SHELXS97* (Sheldrick, 2008 ▶); program(s) used to refine structure: *SHELXL97* (Sheldrick, 2008 ▶); molecular graphics: *SHELXTL* (Sheldrick, 2008 ▶); software used to prepare material for publication: *SHELXTL*.

## Supplementary Material

Crystal structure: contains datablocks global, I. DOI: 10.1107/S1600536809014287/ci2783sup1.cif

Structure factors: contains datablocks I. DOI: 10.1107/S1600536809014287/ci2783Isup2.hkl

Additional supplementary materials:  crystallographic information; 3D view; checkCIF report

## Figures and Tables

**Table 1:** Hydrogen-bond geometry (Å, °)

*D*—H⋯*A*	*D*—H	H⋯*A*	*D*⋯*A*	*D*—H⋯*A*
C12—H12*C*⋯O1^i^	0.96	2.46	3.362 (4)	156 (6)

Symmetry code: (i) 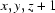.

**Fig. 1.**
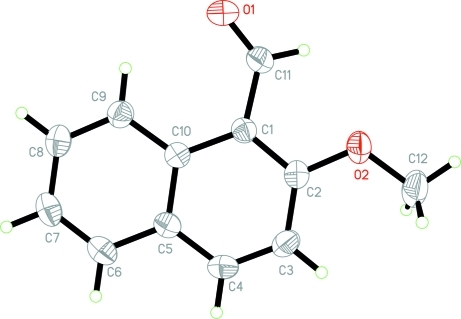
The molecular structure of the title compound, with atom labels and anisotropic displacement ellipsoids (drawn at 50% probability level) for non-H atoms.

**Fig. 2.**
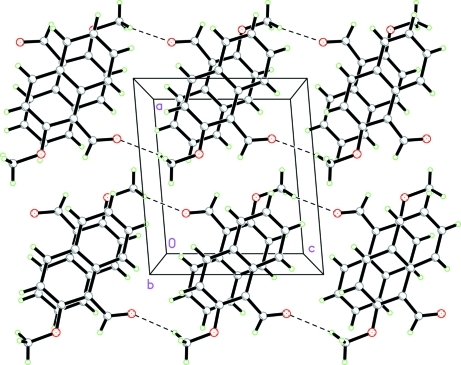
The molecular packing of the title compound, viewed along the b axis. Intermolecular C–H···O hydrogen bonds are shown as dashed lines.

## supplementary crystallographic information

### Comment

A large number of aldehydes were chosen as starting materials for the synthesis of Schiff base derivatives (Yehye *et al.*, 2008; Tabatabaee *et al.*, 2007; Zhang & Li, 2007). We report here the crystal structure of the title compound.

In the title molecule (Fig. 1), the bond lengths are within normal ranges (Allen *et al.*, 1987). The carbonyl oxygen atom O1 deviates from the plane of the naphthalene ring system by 0.027 (2) Å. The aldehyde and methoxy groups are slightly twisted away from the naphthalene ring system [C10—C1—C11—O1 10.6 (3)° and C12—O2—C2—C3 = 8.4 (2)°].

In the crystal structure, molecules are linked through intermolecular C–H···O hydrogen bonds (Table 1), forming chains running along the *c* axis (Fig. 2).

### Experimental

The title compound was obtained commercially (Lancaster). Single crystals suitable for X-ray analysis were obtained by slow evaporation of a methanol solution of the compound.

### Refinement

H atoms were positioned geometrically and refined as riding, with C-H = 0.93–0.96 Å and *U*_iso_(H) = 1.2*U*_eq_(C) and 1.5*U*_eq_(C12).

### Crystal data

**Table:**

C_12_H_10_O_2_	*F*(000) = 392
*M**_r_* = 186.20	*D*_x_ = 1.316 Mg m^−^^3^
Monoclinic, *P*2_1_/*c*	Mo *K*α radiation, λ = 0.71073 Å
Hall symbol: -P 2ybc	Cell parameters from 1550 reflections
*a* = 8.689 (3) Å	θ = 2.3–25.3°
*b* = 14.155 (4) Å	µ = 0.09 mm^−^^1^
*c* = 7.667 (2) Å	*T* = 298 K
β = 94.805 (4)°	Block, colourless
*V* = 939.7 (5) Å^3^	0.20 × 0.20 × 0.18 mm
*Z* = 4	

### Data collection

**Table:**

Bruker SMART CCD area-detector diffractometer	2046 independent reflections
Radiation source: fine-focus sealed tube	1477 reflections with *I* > 2σ(*I*)
graphite	*R*_int_ = 0.018
ω scans	θ_max_ = 27.0°, θ_min_ = 2.4°
Absorption correction: multi-scan (*SADABS*; Sheldrick, 1996)	*h* = −8→11
*T*_min_ = 0.982, *T*_max_ = 0.984	*k* = −17→18
5187 measured reflections	*l* = −8→9

### Refinement

**Table:**

Refinement on *F*^2^	Primary atom site location: structure-invariant direct methods
Least-squares matrix: full	Secondary atom site location: difference Fourier map
*R*[*F*^2^ > 2σ(*F*^2^)] = 0.043	Hydrogen site location: inferred from neighbouring sites
*wR*(*F*^2^) = 0.124	H-atom parameters constrained
*S* = 1.03	*w* = 1/[σ^2^(*F*_o_^2^) + (0.0588*P*)^2^ + 0.1007*P*] where *P* = (*F*_o_^2^ + 2*F*_c_^2^)/3
2046 reflections	(Δ/σ)_max_ = 0.001
128 parameters	Δρ_max_ = 0.12 e Å^−^^3^
0 restraints	Δρ_min_ = −0.17 e Å^−^^3^

### Special details

**Table:**

Geometry. All e.s.d.'s (except the e.s.d. in the dihedral angle between two l.s. planes) are estimated using the full covariance matrix. The cell e.s.d.'s are taken into account individually in the estimation of e.s.d.'s in distances, angles and torsion angles; correlations between e.s.d.'s in cell parameters are only used when they are defined by crystal symmetry. An approximate (isotropic) treatment of cell e.s.d.'s is used for estimating e.s.d.'s involving l.s. planes.
Refinement. Refinement of *F*^2^ against ALL reflections. The weighted *R*-factor *wR* and goodness of fit *S* are based on *F*^2^, conventional *R*-factors *R* are based on *F*, with *F* set to zero for negative *F*^2^. The threshold expression of *F*^2^ > σ(*F*^2^) is used only for calculating *R*-factors(gt) *etc*. and is not relevant to the choice of reflections for refinement. *R*-factors based on *F*^2^ are statistically about twice as large as those based on *F*, and *R*- factors based on ALL data will be even larger.

### Fractional atomic coordinates and isotropic or equivalent isotropic displacement parameters (Å^2^)

**Table:**

	*x*	*y*	*z*	*U*_iso_*/*U*_eq_	
O1	0.28487 (13)	0.04582 (12)	−0.30026 (16)	0.0951 (5)	
O2	0.37442 (12)	0.12897 (9)	0.17417 (14)	0.0716 (4)	
C1	0.17514 (14)	0.10308 (9)	−0.04428 (17)	0.0445 (3)	
C2	0.22015 (16)	0.13172 (9)	0.12545 (18)	0.0493 (3)	
C3	0.11126 (18)	0.16328 (10)	0.23780 (19)	0.0570 (4)	
H3	0.1427	0.1806	0.3523	0.068*	
C4	−0.03974 (18)	0.16829 (10)	0.17843 (19)	0.0561 (4)	
H4	−0.1109	0.1892	0.2539	0.067*	
C5	−0.09270 (15)	0.14282 (9)	0.00617 (18)	0.0467 (3)	
C6	−0.25038 (17)	0.15016 (10)	−0.0548 (2)	0.0596 (4)	
H6	−0.3211	0.1723	0.0200	0.072*	
C7	−0.29984 (17)	0.12527 (11)	−0.2210 (2)	0.0644 (4)	
H7	−0.4038	0.1305	−0.2597	0.077*	
C8	−0.19430 (18)	0.09191 (11)	−0.3336 (2)	0.0617 (4)	
H8	−0.2289	0.0748	−0.4473	0.074*	
C9	−0.04112 (16)	0.08386 (10)	−0.28015 (18)	0.0527 (4)	
H9	0.0269	0.0613	−0.3578	0.063*	
C10	0.01568 (14)	0.10936 (8)	−0.10843 (17)	0.0426 (3)	
C11	0.29521 (17)	0.06484 (12)	−0.1473 (2)	0.0618 (4)	
H11	0.3909	0.0540	−0.0870	0.074*	
C12	0.4284 (2)	0.14661 (15)	0.3509 (2)	0.0850 (6)	
H12A	0.4022	0.2100	0.3816	0.128*	
H12B	0.5385	0.1389	0.3649	0.128*	
H12C	0.3809	0.1029	0.4257	0.128*	

### Atomic displacement parameters (Å^2^)

**Table:**

	*U*^11^	*U*^22^	*U*^33^	*U*^12^	*U*^13^	*U*^23^
O1	0.0651 (8)	0.1637 (14)	0.0584 (8)	0.0113 (7)	0.0161 (6)	−0.0216 (8)
O2	0.0509 (6)	0.1051 (9)	0.0568 (7)	−0.0042 (6)	−0.0083 (5)	−0.0022 (6)
C1	0.0433 (7)	0.0458 (7)	0.0447 (8)	−0.0016 (5)	0.0056 (5)	0.0043 (5)
C2	0.0471 (8)	0.0500 (8)	0.0498 (8)	−0.0030 (6)	−0.0009 (6)	0.0033 (6)
C3	0.0680 (10)	0.0567 (8)	0.0457 (8)	0.0016 (7)	0.0013 (7)	−0.0087 (6)
C4	0.0613 (9)	0.0534 (8)	0.0555 (9)	0.0088 (6)	0.0156 (7)	−0.0047 (6)
C5	0.0475 (7)	0.0399 (7)	0.0533 (8)	0.0015 (5)	0.0082 (6)	0.0027 (6)
C6	0.0469 (8)	0.0604 (9)	0.0727 (11)	0.0051 (6)	0.0122 (7)	0.0066 (7)
C7	0.0431 (8)	0.0705 (10)	0.0782 (12)	−0.0019 (7)	−0.0039 (7)	0.0090 (8)
C8	0.0575 (9)	0.0658 (9)	0.0595 (10)	−0.0065 (7)	−0.0090 (7)	−0.0017 (7)
C9	0.0523 (8)	0.0546 (8)	0.0508 (8)	−0.0024 (6)	0.0028 (6)	−0.0024 (6)
C10	0.0453 (7)	0.0372 (6)	0.0456 (8)	−0.0018 (5)	0.0045 (5)	0.0035 (5)
C11	0.0463 (8)	0.0847 (11)	0.0552 (9)	0.0004 (7)	0.0089 (6)	0.0020 (8)
C12	0.0713 (11)	0.1163 (16)	0.0632 (11)	−0.0073 (10)	−0.0195 (9)	−0.0018 (10)

### Geometric parameters (Å, °)

**Table:**

O1—C11	1.1991 (18)	C6—C7	1.357 (2)
O2—C2	1.3615 (16)	C6—H6	0.93
O2—C12	1.4180 (19)	C7—C8	1.393 (2)
C1—C2	1.3877 (19)	C7—H7	0.93
C1—C10	1.4336 (18)	C8—C9	1.364 (2)
C1—C11	1.4641 (19)	C8—H8	0.93
C2—C3	1.405 (2)	C9—C10	1.4137 (19)
C3—C4	1.354 (2)	C9—H9	0.93
C3—H3	0.93	C11—H11	0.93
C4—C5	1.409 (2)	C12—H12A	0.96
C4—H4	0.93	C12—H12B	0.96
C5—C6	1.414 (2)	C12—H12C	0.96
C5—C10	1.4219 (19)		
			
C2—O2—C12	119.74 (13)	C6—C7—H7	120.1
C2—C1—C10	119.47 (12)	C8—C7—H7	120.1
C2—C1—C11	117.16 (12)	C9—C8—C7	121.22 (14)
C10—C1—C11	123.34 (12)	C9—C8—H8	119.4
O2—C2—C1	116.27 (12)	C7—C8—H8	119.4
O2—C2—C3	122.61 (13)	C8—C9—C10	120.94 (14)
C1—C2—C3	121.11 (13)	C8—C9—H9	119.5
C4—C3—C2	119.60 (13)	C10—C9—H9	119.5
C4—C3—H3	120.2	C9—C10—C5	117.58 (12)
C2—C3—H3	120.2	C9—C10—C1	123.75 (12)
C3—C4—C5	122.21 (13)	C5—C10—C1	118.67 (12)
C3—C4—H4	118.9	O1—C11—C1	127.75 (15)
C5—C4—H4	118.9	O1—C11—H11	116.1
C4—C5—C6	121.50 (13)	C1—C11—H11	116.1
C4—C5—C10	118.91 (13)	O2—C12—H12A	109.5
C6—C5—C10	119.59 (13)	O2—C12—H12B	109.5
C7—C6—C5	120.87 (14)	H12A—C12—H12B	109.5
C7—C6—H6	119.6	O2—C12—H12C	109.5
C5—C6—H6	119.6	H12A—C12—H12C	109.5
C6—C7—C8	119.79 (14)	H12B—C12—H12C	109.5

### Hydrogen-bond geometry (Å, °)

**Table:**

*D*—H···*A*	*D*—H	H···*A*	*D*···*A*	*D*—H···*A*
C12—H12C···O1^i^	0.96	2.46	3.362 (4)	156 (6)

Symmetry codes: (i) *x*, *y*, *z*+1.
